# Translating evidence into practice: a longitudinal qualitative exploration of allied health decision-making

**DOI:** 10.1186/s12961-020-00662-1

**Published:** 2021-03-18

**Authors:** Jennifer White, Kellie Grant, Mitchell Sarkies, Terrence Haines, Evidence Translation in Allied Health (EviTAH) Group, Meg E. Morris, Leeanne Carey, Nicholas F. Taylor, Anne E. Holland, Anne Bardoel, Cylie Williams, Lisa O’Brien, Elizabeth H. Skinner, Jenny Martin

**Affiliations:** 1 University of Newcastle, University Drive, Callaghan New South Wales, Newcastle, New South Wales Australia; 2 School of Primary and Allied Health, Monash University, Frankston, VIC Australia; 3 Curtin University, Perth, WA Australia

**Keywords:** Allied health, Evidence-based practice, Implementation science, Qualitative

## Abstract

**Background:**

Health policy and management decisions rarely reflect research evidence. As part of a broader randomized controlled study exploring implementation science strategies we examined how allied health managers respond to two distinct recommendations and the evidence that supports them.

**Methods:**

A qualitative study nested in a larger randomized controlled trial. Allied health managers across Australia and New Zealand who were responsible for weekend allied health resource allocation decisions towards the provision of inpatient service to acute general medical and surgical wards, and subacute rehabilitation wards were eligible for inclusion. Consenting participants were randomized to (1) control group or (2) implementation group 1, which received an evidence-based policy recommendation document guiding weekend allied health resource allocation decisions, or (3) implementation group 2, which received the same policy recommendation document guiding weekend allied health resource allocation decisions with support from a knowledge broker. As part of the trial, serial focus groups were conducted with a sample of over 80 allied health managers recruited to implementation group 2 only. A total 17 health services participated in serial focus groups according to their allocated randomization wave, over a 12-month study period. The primary outcome was participant perceptions and data were analysed using an inductive thematic approach with constant comparison. Thematic saturation was achieved.

**Results:**

Five key themes emerged: (1) Local data is more influential than external evidence; (2) How good is the evidence and does it apply to us? (3) It is difficult to change things; (4) Historically that is how we have done things; and (5) What if we get complaints?

**Conclusions:**

This study explored implementation of strategies to bridge gaps in evidence-informed decision-making. Results provide insight into barriers, which prevent the implementation of evidence-based practice from fully and successfully occurring, such as attitudes towards evidence, limited skills in critical appraisal, and lack of authority to promote change. In addition, strategies are needed to manage the risk of confirmation biases in decision-making processes.

*Trial registration* This trial is registered with the Australian New Zealand Clinical Trials Registry (ANZCTR) (ACTRN12618000029291). Universal Trial Number (UTN): U1111-1205-2621.

## Introduction

There is a global move towards the implementation of science-led healthcare practices, commonly referred to as evidence-based practice. Evidence-based practice is underpinned by research and guided by principles of safety, effectiveness, person-centred care, timeliness, efficiency, and equity [1] alongside clinical expertise, and the values of people who seek healthcare [2]. There has also been an exponential growth in the number of systematic review findings which places demands on clinicians for analysis and comprehension of healthcare evidence [3, 4]. However, finite resources mean that allied health managers must make explicit or implicit choices about how to prioritize interventions, funds and staff time.

Failure to translate research findings into day-to-day healthcare practices results in evidence-based practice gaps [5]. Bridging the gaps to implement the best available and most relevant evidence is a major challenge for the health system. A recent study in Australia suggested that on average 57% of persons in healthcare settings receive care according to evidence-based guidelines [6]. A similar earlier study in the USA demonstrated concordant results with almost 55% of patients being provided with recommended care consistent with evidence [7]. There remains many possible reasons why evidence-based practice gaps continue to exist [8]. Health professionals have been reported as not engaging in evidence-based practice activities because they do not see these as core components in clinical care [9]. Further, health professionals may lack skills towards searching and evaluating research findings. In addition, the impact of increasing caseloads is cited as a barrier to accessing necessary databases [9–11]. Despite limitations in the current application of evidence-based practice, it is still reported to be an important issue amongst health professionals [10, 11].

Evidence-informed decision-making refers to the complex process of considering the best available evidence when planning, evaluating and delivering health services [12–14]. High quality research, professional experience, expert opinion, values of people receiving healthcare services, and local contextual factors may all contribute to the evidence-informed decision-making process [15]. However, organizational resistance to change can provide an internal barrier to evidence-informed decision-making [16], where external limitations such as lack of financial incentives, community views, and political context create a complex external environment to navigate [17–19]. Difficulties in making choices have been well described in marketing research, especially when a decisions involve trade-offs [20]. However, there is increasing interest in the influence of cognitive and affective biases in medical and allied health decision-making [20, 21]. For example, *availability heuristic bias* has been described in medical decision-making and pertains to whether a person can retrieve information from their mind to make a decision [22, 23]. *Loss/gain framing bias* involves a tendency to focus on potential losses when considering a decision [24]. *Sunk cost bias* represents the impact of irrevocable past decisions on future decision-making [25]. However few studies explore allied health decision-making beyond psychology and these typically explore clinical rather than resource allocation decision-making [26]. Indeed, little is known about how allied heath managers make decisions regarding resource allocation [27].

Central to the context of this study is evidence that, within Australia, allied health therapeutic services are generally provided during hospital business hours [28, 29]. Typically, reduced allied health services are available during weekends, with the goal of facilitating patient discharge, and preventing adverse events and escalation of care. Further, there is a variety in weekend allied health provision both within [30] and between health services [28, 29]. Within services, allied health departments have different levels of availability during weekends based on perceived benefits [31, 32]. Between services, there is a large unwarranted variation in care, with approximately 43–61% of acute hospitals and 30–53% of subacute hospitals providing weekend services [28, 29]. Variations in care can often be ascribed to uncertainty around the effectiveness of specific healthcare approaches [33], emphasizing the importance of evidence dissemination and implementation into policy and practice.

Research into existing evidence–practice gaps in areas specifically relevant to allied health disciplines is limited. This is important as more than 25% of the Australian healthcare workforce are allied health professionals, with an estimated 195 000 clinicians delivering 200 million episodes of care annually [34, 35]. Together with doctors and nurses, allied health professionals play a vital role among healthcare providers in Australia, providing interventions that are essential to the functioning of an efficient and effective health system [35]. The aim of this research was to explore the experience of decision-making in allied health managers towards making practice change based on access to a policy recommendation document.

## Method

### Design

Qualitative social research investigates the relationships between individuals and the institutions and society in which they live [36]. This prospective qualitative study involved serial focus groups with allied health managers [37] and was informed by the Consolidated criteria for reporting qualitative research (COREQ) checklist [38]. Participants were recruited as part of larger study exploring research implementation strategies from Australian and New Zealand hospitals, reported elsewhere [39]. As part of the larger study participants were randomized to (1) control group or (2) implementation group 1, which received an evidence-based policy recommendation document guiding weekend allied health resource allocation decisions [40], or (3) implementation group 2, which received the same policy recommendation document guiding weekend allied health resource allocation decisions with support from a knowledge broker. The primary outcome was alignment of weekend allied health service provision with policy recommendations at 12-month follow-up. This qualitative substudy explored secondary outcomes regarding the experience of decision-making among allied health managers, randomly allocated to implementation group 2. Focus groups occurred between June 2018 and September 2019 and all participants provided written, informed consent. The Monash Health Human Research Ethics Committee approved the study (Res-17-0000-067L).

### Participants and setting

Allied health managers across Australia and New Zealand who were responsible for weekend allied health resource allocation decisions towards the provision of inpatient service to acute general medical and surgical wards, and subacute rehabilitation wards were eligible for inclusion. The process of recruitment and randomization is described elsewhere [39].

### Procedure

Participants in the broader study, involving a three-group matched (based on health service regional status) parallel cluster randomized controlled trial [39], were randomized at varying times depending on acquiring multisite ethics approval, time of consent and collection of baseline data forming a convenience sample. In total eight waves (groups) of participants were recruitment between May 2018 and September 2019, thus resulting in six groups of participants who engaged with the knowledge broker (JW), an experienced occupational therapist, department manager and researcher. Participants and the number of sites varied between each wave (Table 1). While participants were encouraged to consider the evidence and recommendations [40], there was no obligation for participants to change weekend allied health service delivery or engage with the knowledge broker.

**Table 1:** Randomization, and access to support from a knowledge broker

Wave	Potential participants	Number of sites	Number of focus groups with knowledge broker
1	55	4	5
2	23	3	2
3	35	3	2
4	49	4	3
7	15	1	3
8	7	2	2

### Data collection

Focus groups were conducted via teleconference, ideally, where video allowed, and promoting eye contact throughout. The knowledge broker, an experienced qualitative researcher (JW, described above), conducted the focus groups and one facilitator, a social worker (KG), took detailed field notes [41] that informed continued data analysis. At the time of the focus group participants were briefed about the researcher and the project. The knowledge broker (JW) was not involved in participant randomization and this assisted to reduce bias throughout the focus groups; however, she did invite participation and introduce herself as the knowledge broker. Out of a total of six randomized waves of participants, up to five focus groups were conducted within each wave during the 12-month study period (see Table 1). The number of participants who attended focus groups varied and a focus group only proceeded when two or more participants had responded to indicate that they would participate because of the discussion-based nature of focus group methodology. Higher numbers of participants (range 6–18) attended the first focus group in which the knowledge broker presented results from the systematic review of literature towards weekend allied health [39] and recommendations, as outlined in the evidence-based policy recommendation document [40]. A topic guide was used during focus groups and questions were open-ended and focused on participants’ reactions to the presented evidence and how they considered they might respond (Table 2). Ongoing focus groups allowed for a deeper exploration of the evidence, barriers and facilitators to practice change and previous experiences of making practice changes. Consequently, the participants contributed as much detailed information as they wished, and the researchers asked further questions as necessary towards barriers, facilitators, and processes that affected making practice changes.

**Table 2:** Interview guide

Questions	Probe
What is your initial reaction to the recommendation? Expand	What did you think of the information presented? How do feel after listening to this presentation? Do you feel that the evidence-based policy recommendation is applicable to your health service? Why/Why not? Do you feel that the results presented within individual papers have been reported in a trustworthy manner by the authors? (Why/Why not) Do you feel that the approach taken by the EviTAH investigators to identify and synthesize this information has been trustworthy? (Why/Why not)
Do you feel that the evidence synthesized to form the evidence-based policy recommendation is applicable to your health service? (Why/Why not)	Do you feel that the recommendations formed by the EviTAH investigators accurately reflects the evidence that was identified? (Why/Why not) Do you have any concerns? What do you have particular concerns about? Length of stay, outcomes—reflect on how they perceive the evidence and its meaning to their setting
What do you perceive the recommendations mean towards the provision of your acute and subacute services?	Are you concerned that there may be risks associated with implementing the recommendation? What are some of these? What leads you to believe these may happen? (source of information, personal perception) Is there anything else that you think will be important towards implementing the recommendations?
What did you think of the information presented?	What do you see as your role in terms of ensuring that resources used in allied health are done so in a way that maximize benefit to the community?
At this stage how do you feel you will respond?	What are the main reasons that you intend to respond or not? What do the recommendations mean for your acute service delivery? If you already think your acute services are provided on the basis of clinical exception, what are the exceptions? What do the recommendations mean for your subacute service delivery?
Does the make-up of your wards align with the results	How/how not? Clarify re geriatric evaluation and management Do not know what to do? Explore current service in comparison to evidence-based policy recommendation
What does the evidence mean to your service delivery?	Anticipate barriers, for example time, effort, specialist Is there anything else that you think will be important towards implementing the recommendations?
Are you completely accepting of evidence-based policy recommendation? Expand	
Are you accepting but not willing to act?	Why?
What do you see as barriers that could be overcome?	How could these be overcome? Explore barriers perceived as resolvable and those that are not Perceived concerns/risks?

### Data analysis

All focus groups were recorded and transcribed verbatim. The primary author checked transcripts for accuracy. Two experienced qualitative researchers (JW and KG) conducted data analysis guided by an inductive thematic approach [42]. Analysis involved constant comparison, concurrent data collection and analysis (whereby analysis informed data collection in further interviews) [43]. The researchers sequentially examined each transcript, both in relation to emerging codes and concepts, in the cohort and in temporal relationship by same health service participants. Each researcher sustained engagement with the data through an initial reading and re-reading of transcripts to identify units of meaning and initial codes. We acknowledged participant silence to look for the implicit meanings within participant responses [43]. Following team discussion initial codes were used to identify key categories and the primary author merged codes into a single document. At this stage, both researchers re-read the previously coded transcript alongside the identified categories, in order to ensure that these appropriately fitted the original text. In the final step, emerging categories were refined and grouped together into themes with input from the broader team. Rigour was upheld through immersion in data, reflexive analysis, and peer debriefing [44]. In addition, consensus coding between team members from different professional backgrounds (JW an occupational therapist, KG a social worker and TH a physiotherapist) followed by discussion with the broader team addressed the potential for bias [44]. Coders also captured exemplar quotes supporting each theme. Member checking was not undertaken to reduce the data collection burden for time-constrained managers.

## Results

In total, 89 participants from 17 health services took part in the 17 focus groups (ranging from 1 to 2 h), according to their allocated randomization wave. The initial focus groups received greater levels of participation (range 6–18) compared with the follow-up focus groups (range 2–8). Only two services engaged in more than two sessions with the knowledge broker. The most common reason not to participate further was the desire to make an internal decision. Two health services did not engage with the knowledge broker and did not provide a reason. Participant demographics for the focus groups are shown in Table 3. Participants were mostly female and included managers from occupational therapy and physiotherapy professional backgrounds, likely due to the nature of the recommendations focusing on these professions.

**Table 3:** Participant demographics

Region	Number (%)
Rural	50 (56)
Metropolitan	36 (40)
Mixed	2 (4)
Ward type	
Acute	73 (82)
Subacute	8 (1)
Mixed	6 (5)
Discipline	
Dietician	12 (13)
Nursing	5 (1)
Occupational therapy	16 (18)
Podiatry	6 (1)
Physiotherapy	17 (19)
Speech pathology	12 (13)
Social worker	13 (1)
Other	14 (16)
Qualification	
Bachelors	34 (38)
Doctorate	1 (3)
Honours	23 (26)
Masters	29 (33)

Discussion among participants across the serial focus groups provided insights into resource allocation and decision-making, including the interplay between barriers and facilitators concerning implementation of recommendations outlined in the evidence-based policy recommendation document. Five key themes emerged:

1. Local data is more influential than external evidence.
2. How good is the evidence and does it apply to us and our patients?
3. It is difficult to change things.
4. Historically that is how we have done things.
5. What if we get complaints?

1. Local data is more influential than external evidence

There were similarities across each wave of focus groups towards participant understanding of reading and interpreting research evidence. Only a few participants had a research or academic background and therefore the nature of participant questions, the silence of participants or hesitation towards interpreting the systematic review appeared to reflect a lack of understanding, and confidence in understanding research data. For example, participants asked questions about the outcome measures reported in the included studies and felt more should be included. They also expressed concern about why studies were included and questioned the timing of included studies.

Your research was before 12 months ago. How does that apply to the orthopaedic ward that is in practice today? (wave (W) 2, session (S) 1)

Specifically, there appeared to be limited understanding about statistical interpretation. One experienced researcher questioned the process used towards the systematic reviews.

I don’t think that as far as policy or any other document [is concerned can be]… based on two factors, one favours the intervention, one favours the control. Overall, it just is non-systematically done. (W1, S1)

Most participants reported to be more confident in results if there were a larger number of studies supporting an outcome, in contrast to the sample size, effect size, and confidence interval reported within a given study. As a result, participants reported there was “Insufficient data” (W6, S2) and quality of included studies, such as “Patchy evidence” (W1, S1) to support the research findings. This was closely linked with a reluctance to consider changing the status quo.

What we are saying is there is not a great number of studies. Yes, there is pooled results, but this is based on still a very limited number of studies. (W5, S2)

I guess we do not feel that there is enough evidence out there to make a change. (W2, S2)

Most participants cited a preference for making decisions according to studies they had previously relied on to develop their current weekend clinical protocols. All these studies advocated for weekend allied health, such as early mobilization post surgery. However, these studies were not included in the systematic review (criteria cited elsewhere [39]).

Surgery is an interesting one, because there is evidence for it outside of the weekend service. There is actually evidence out there with regard to early mobilization, for a fractured knee, the knee replacement…. I am inclined to say we should stick with the protocols we have got with that regards. I would not be changing anything there. (W1, S2)

### Internal data reliance

After reviewing the results of the systematic review, participants reported an overwhelming response for the need to revisit internal, local health service data. Subsequent participant reports suggested that internal data was the primary source of information for consideration when making resource allocation decisions.

The lack of evidence [in the systematic reviews] does not mean that we should not be providing that service; we just do not have enough evidence… so we deal with our own [internal] evidence. We know what works in our site and have that information. (W1, S2)

Other participants indicated that they preferred to verify the results for themselves before making decisions. This reportedly involved revisiting the papers in the systematic review, as well as reflecting on internal data.

I think for me, I need to go back, re-read the document. I need to also go back and look at some of the data [internal] that I have around… look at some of the files to see exactly what has happened. (W7, S1)

Alternatively, participants reported hearing that there were studies currently in progress promoting weekend allied health services. As a result, some participants preferred to wait for these findings to be published. As such, participants appeared to place value in incomplete research over and above completed work.

From my understanding, there is a very positive trial happening. I do not remember what they are looking at. But they think they have actually increased orthopaedic services [on the weekend], in response. (W3, S1)

Closely linked with a reliance on descriptive local knowledge when making resources decisions, was a reliance on collective data, such as benchmarking.

What about what other sites are doing?… is there benchmarking data? (W4, S1)

### Personal values and anecdotal reports

An additional factor potentially driving respondents to prioritize local data and other forms of evidence, not included in the systematic review, was that the findings and recommendations were often not congruent with participants’ reported beliefs, values and wishes for their practice. Where the evidence conformed to their beliefs, the evidence was more enthusiastically accepted.

In regard to the research around the subacute setting, we would absolutely love to provide some sort of weekend service. (W1, S3)

Participants also strongly relied upon anecdotal staff experiences. Participants indicated suggested that their colleagues felt that weekend allied health services facilitated flow of people through the healthcare service, discharge, and workload pressures, while ensuring care.

To actually churn things through [move patients] is, I suspect, why we have the weekend staff at the end of the day. (W5, S2)

Overall participants readily stated that local context was a key factor informing decision-making and these were based on internal reviews of their weekend service.

We have moved services around and have a number of proposals [for weekend allied health services] ready to go. These are based on evidence so to speak—of the actual work that we have been doing. (W1, S3)

2. How good is the evidence and does it apply to us and our patients?

Participant discussions often expressed the perception that patient experience was more important than the evidence, such as patient feedback or satisfaction reports from people receiving their healthcare services. At times, this appeared to be valued over and above published evidence. For example, participants stated that patient comfort, satisfaction and desire for regular contact with a therapist were integral to decision-making about what services were needed.

Anecdotal data on patient comfort, you know things like that… that is what we rely on. (W2, S1)

Participants working in private settings most commonly cited the importance of patient feedback in decision-making.

You know, I have been in private heath now, our clients are, our customers are fairly demanding. I would argue more so than when I was in public health. (W2, S2)

Other frequently cited local contextual issues were the expectations of medical and nursing staff. Many participants reported that surgeon expectations influenced allied health service delivery and, as such, they felt curtailed in what they could change. Despite the availability of skilled nurses, many sites reported that there was an expectation of allied health providing key interventions, such as mobilizing or assisting with activities of daily living. In many health services, this informed nursing roles and staff rosters; thus, to make changes reportedly required a cultural shift underpinned by extensive consultation and education.

Now, I hear what you are saying, that things do not necessarily have to be done by a physiotherapist. But often there are barriers that come up if it [a service] is not instigated by allied health—depending on their local culture strength. (W3, S2)

Overall, upper level managers’ cited data was only one of many components that informed decision-making, such as staff availability, managing resources and system processes.

I think the complexity for managers is that published evidence is one source of data, but it is not the only source of data in which we make decisions. We draw on a systems-based approach to thinking about how services are delivered, whom they are delivered to, how we have to manipulate and be flexible with resources. Both parts of the puzzle are not necessarily things that you will go to a formal piece of evidence to look at, because it is a dynamic decision-making process. Does that make sense? (W1, S3)

3. It is difficult to change things

Participant reports highlighted their hesitancy in responding to recommendations and deciding. A key reported barrier towards implementing evidence was that participants anticipated that additional, future conflicting evidence would emerge in support of weekend allied health service delivery.

Because I don’t need to find out later that someone comes out with a really fantastic control trial in two years… proving equivocally that occupational therapists and physiotherapists get people out much quicker if they see them on a weekend. (W2, S1)

Another challenge expressed by participants was balancing the research evidence with what they believed to be high quality, individualized care, even if data was not available to support these beliefs.

Out of all of those, I think most clinicians probably say function and quality of life is what they are hoping to change. I do not think people go into health so they can reduce length of stay. (W2, S3)

In the absence of sufficient evidence, participants placed significant value in their clinical reasoning and the perceived effectiveness of services provided by their profession.

Yeah, just without the evidence, we are going to rely on our own reasoning, I guess, and that is what we have done. (W3, S1)

Given that most studies in the systematic review pertained to occupational therapy and physiotherapy services on the weekend, participants from other allied health disciplines expressed an interest in undertaking research or publishing existing, local research in order to advocate for their profession.

It is not public, but I tell you what, you really encouraged me to publish. This has really lit a fire in my belly to do some research and publish [local data] on this stuff. (W1, S1)

Overall, where there was more evidence, there was more willingness to act on the recommendation.

I think our sense is probably we will be looking at the physiotherapy and occupational therapy recommendations where there are sort of more evidence taken. (W4, S1)

Participants reported that components of their weekend services were often funded by different divisions whereby there was the expectation to provide weekend allied health. While making a change was not considered insurmountable, it was perceived to be difficult. As such, there was a preference among participants to continue meeting the expectations of their funders.

We could move the staff… but the difficulty comes when you have been allocated specific funding by a specific part of the organization. I am not saying that it cannot be done, but certain parts of the service do allocate the funds. (W1, S2)

We provide professional supervision for them; so, we can still influence [service provision] but we cannot actually make the decisions to change weekend staffing. (W2, S3)

Likewise, some participants reported confusion about the perceived variances in models of care across the Australian states and this impacted decision-making towards making a service change. For example, there was a perceived lack of subacute services in New South Wales providing the impetus for more acute services, including weekend allied health. As a result, important local contextual differences influenced allied health manager decision-making.

We do not have a very good understanding of models of care across Australia and how they are similar or different. Yet we are being asked to make decisions on evidence from some studies, not looking at the whole picture. (W5, S1)

Interestingly for rural and regional services, the lack of allied health on the weekend was routine practice. By default, they aligned with the recommendations but also explored other options such as “Giving iPads to our patients so they can do self-directed therapy on the weekends” (W4, S1). However, metropolitan sites appeared reluctant to reflect on this variance, despite consistency with study recommendations. Instead, metropolitan participants remained silent at this point in the focus group.

4. Historically that is how we have done things

Participants who were reluctant to change weekend allied health service delivery cited a belief in the perceived benefit of historical weekend allied practice, particularly when considering the need for allied health to respond to the timing of orthopaedic surgery.

I mean it [the recommendations] goes against the physiotherapist. And obviously we have a long tradition of providing quite extensive services to acute medical and surgical wards. (W1, S1)

We have acute care cover for the elective surgical patients on the weekend so that we can meet their requirements with most surgeries done on Thursday and Friday. (W2, S3).

When considering relocating staff in response to the recommendations, many participants reportedly feared that any weekend allied funding would be lost and redistributed to services other than allied health.

It will take us more than 10 years to try and find that funding again—you know what I mean? We are going to shoot ourselves in the foot. (W1, S1)

Participants also reflected on the past time and effort required to successfully advocate for a weekend allied service delivery on the weekend. As a result, considering making changes was difficult and participants feared that a change in service would reflect poorly on the allied health service reputation.

We worked so hard across disciplines—to actually get allied health as a recognized part of care over the weekend. (W3, S2)

5. What if we get complaints?

The majority of participants reported that the fear of receiving complaints was a major barrier to modifying their weekend allied health service model. The most commonly cited reason was the long-standing expectation in providing a weekend service.

I think we have a lot of pushbacks from our facility if we even stop to talk about stopping weekend services that we currently provide. (W2, S2)

Enormous amount of noise [complaints]. You will also potentially get complaints from patient and patient’s family. (W1, S2)

Participant reports also suggested that some recommendations placed patients at risk of complication if they were not seen on the weekend. This had the potential to create additional costs for the organization.

As a dietitian, it is just appalling that someone would be left [nil by mouth] for 72 h [over the weekend]. What you are going to have to do is put in a drip, and all the cost associated with that. So, even though there may not be evidence, I think that’s a pretty big, common-sense element that comes in to have an assessment from a speech pathologist, rather than having to wait…. (W1, SA1)

A lot of our joint replacement patients are going home day two and if day two falls on a weekend—we are going to miss them. We need to see and review them and make sure everything is in place for when they go home. (W3, S2)

Dealing with complaints was perceived to be a time-consuming process and therefore efforts were made to pre-empt and avoid complaints wherever possible. As such, when participants anticipated complaints in response to toward making staff change to a well-established service delivery model, they cited that they needed to “Do our homework” (W6, S2) to be certain before making a change.

Perceived changes in society towards a 7-day workweek was another commonly cited variance towards the need to maintain weekend allied health service delivery. Indeed, having 7-day allied health was viewed as favourable considering society expectations, such as those observed in the retail industry.

It is becoming almost archaic now: the notion of a weekend and will definitely be so in 10 years’ time. (W3, S1)

## Discussion

Where the evidence aligned with prior expectations and previous decisions, allied health managers appeared agreeable to the recommendations, with only local contextual factors and resourcing appearing to stand in the way of adoption. Where the evidence did not align, there were several themes identified describing what would likely be barriers to implementation of the recommendation.

Our results add valuable information towards resource allocation decision-making in allied health. Extensive literature highlights the presence of bias in clinical decision-making in allied health practice and the likelihood of inconsistencies and potential for suboptimal outcomes [21, 26, 45]. Indeed, decision-making remains a complex and multifaceted process [45] and gaps in our understanding continue to exist since most research pertains to clinical decision-making in medical [23] and psychology professions [26] or clinical errors [23, 45]. Our findings contribute to existing knowledge of allied health resource allocation decision-making and we identified a key barrier that revolved around acceptance of evidence not aligning with prior expectations. When decision-making in this context was discussed, participants indicated that they would turn towards local data, benchmarking data, and anecdotal reports from staff and people receiving care. Internal inconsistency was noted, as was the inconsistency with established frameworks for forming recommendations based on research evidence (such as the GRADE criteria used by the Cochrane Collaboration [46]). These observations may be symptomatic of confirmation bias, which has been observed in many fields when evidence is presented that does not conform to prior expectations and beliefs [47] and affective bias, where a person’s emotional state influences decision outcomes [26]. Similarly, sunk cost bias was evident in that participants were more inclined to weekend service provision as a result of previously invested resources [48]. This has implications for future research seeking to assist allied health decision-makers to use evidence conflicting with prior expectations and decisions. Strategies are needed to help decision-makers to internally manage the risk of confirmation biases distorting how they go about their decision-making processes.

A second barrier existed when evidence was lacking for specific allied health disciplines. Recent research suggests that resource allocation decisions are made difficult by a paucity of research evidence [27] and the desire for equity [49]. Our study identified that participants continued advocating for services on the basis of the underlying effectiveness belief, until evidence proved contrary. This appears to be in reversal of the burden of proof principle that requires sufficient evidence be provided to support a given practice [50]. In addition recency bias may have an effect when participants reflect how recently information has been obtained [22]. Indeed allied health managers themselves described an absence of evidence, but continued advocacy for weekend services [51]. This culture of burden of proof reversal, where services are implemented despite a lack of evidence, exists across many Australian and New Zealand health services.

This study highlights that implementing an evidence-based public health service is complex. While some proponents of evidence-based practice adopt greater specificity, such as GRADE components, others are less clear, which means it is difficult to make decisions especially when local contextual factors exist. Driving change in healthcare commonly encounters resistance and scepticism. Providing leadership development skills has the potential to equip allied health leaders and reduce feelings of disempowerment and frustration. Overall, changing the status quo can be timely and costly process due to the need to identify where resistance exists, create awareness and address any cognitive bias. Further, this may involve incorporating psychological indicators to help overcome barriers and carefully plan proposed changes [52].

### Strengths and limitations

The strength of this study lies in the longitudinal, qualitative methodology and tracking of changes over time. A broad range of experiences were identified, and we achieved thematic saturation; however, we did not explore issues affecting allied health decision in the other implementation groups and such understandings would provide further insight into how changes are made. However, our results provide emerging insights into allied decision-making. This is important since allied health is often arranged in discipline-specific departments that need to service multiple clinical areas [53] and make frequent choices between allocating resources to different clinical areas. A suggestion for future research would be to conduct a pre–post survey of all participant attitudes and knowledge to allow for greater comparison. We acknowledge a barrier in the use of videoconferencing and future studies may result in improved engagement and adoption of recommendations if resources are made available to allow knowledge brokers to travel and meet participants in person. Results may not be transferable to other countries where there are inherent funding differences.

## Conclusion

Results from this study provide insight into barriers, which prevent the implementation of evidence-based practice from fully and successfully occurring, such as attitudes towards evidence, limited skills in critical appraisal, and lack of authority to promote change. Although there is a growing body of knowledge, there remain many unanswered questions towards translational science. What works in one context of care may or may not work in another setting, suggesting that context variables matter in implementation.

## Data Availability

The qualitative data used and/or analysed during the current study are available from the corresponding author on reasonable request.

## Abbreviations

S: Session
W: Wave
